# Supplementary material for: Metabolism-dependent secondary effect of anti-MAPK cancer therapy on DNA repair
Source: NAR Cancer. 2024 Apr 30;6(2):zcae019. doi: 10.1093/narcan/zcae019 (PMC11059277; doi:10.1093/narcan/zcae019)
Supplement: zcae019_Supplemental_Files [file zcae019_supplemental_files.zip › supplementary table legends.docx]

Supplementary table legends

**Supplementary Table S1**

Fig1_DESeq_MAPKi_VS_DMSO_24h: List of genes regulated by MAPKi treatment for 24h.

Fig2_MAPKi_TDD_table: List of mRNAs those TDD is regulated by MAPKi treatment.

Fig3_CC_peaks: List of mRNAs containing ribosomal peaks in control cells.

Fig3_TC_peaks: List of mRNAs containing ribosomal peaks in MAPKi-treated cells.

Fig3_permutation_stats_peaks: statistical analysis comparing amino acid- or codon-composition of ribosomal pics in control- and MAPKi-treated cells.

Fig4_-Q_TDD_table: List of mRNAs those TDD is regulated by glutamine-depletion.

Fig4_CTU2_TDD_table: List of mRNAs those TDD is regulated by CTU2 depletion.

Fig4_permutation_stats_-Q_peaks: statistical analysis comparing amino acid- or codon-composition of ribosomal pics in control- and Q depletion conditions.

Fig4_DESeq_siCTU2_VS_siGL2: List of genes regulated by CTU2 depletion.

Fig4_DESeq_siEPRS1-Q_VS_siGL2: List of genes regulated by EPRS1 depletion.

Fig4_DESeq_siGL2-Q_VS_siGL2: List of genes regulated by glutamine depletion.

Fig5_DESeq_MAPKi_2D_VS_DMSO: List of genes regulated following 2 days recovery after MAPKi treatment.

**Supplementary Table S2**

siRNA: Sequences of siRNAs

primers: Sequences of primers

antibodies: commercial references for antibodies
