# Supplementary material for: Metabolism-dependent secondary effect of anti-MAPK cancer therapy on DNA repair
Source: NAR Cancer. 2024 Apr 30;6(2):zcae019. doi: 10.1093/narcan/zcae019 (PMC11059277; doi:10.1093/narcan/zcae019)
Supplement: zcae019_Supplemental_Files [file zcae019_supplemental_files.zip › Sup_Figures_revised_v2.pptx]

## Slide 1
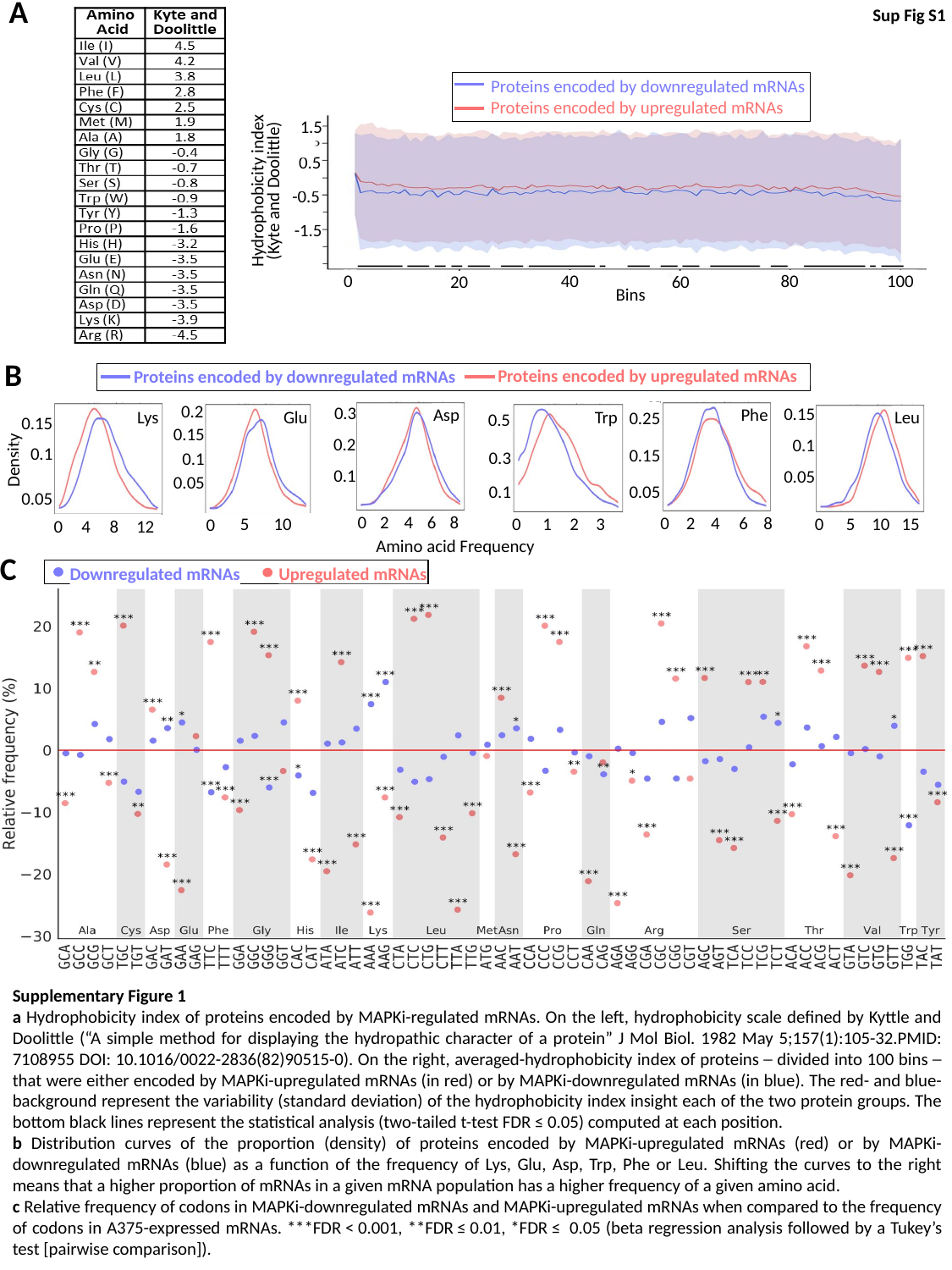

A
Sup Fig S1
Proteins encoded by downregulated mRNAs
Proteins encoded by upregulated mRNAs
0
100
80
40
20
60
Bins
1.5
0.5
Hydrophobicity index
(Kyte and Doolittle)
-0.5
-1.5
B
Proteins encoded by upregulated mRNAs
Proteins encoded by downregulated mRNAs
0.2
0.3
Asp
Lys
Glu
0.15
0.15
0.2
0.1
0.1
Density
0.1
0.05
0.05
8
2
0
4
6
10
5
0
0
4
8
12
0.15
Phe
Leu
Trp
0.25
0.5
0.1
0.15
0.3
0.05
0.1
0.05
2
0
4
6
8
2
3
0
5
10
15
0
1
Amino acid Frequency
C
Upregulated mRNAs
Downregulated mRNAs
Supplementary Figure 1
a Hydrophobicity index of proteins encoded by MAPKi-regulated mRNAs. On the left, hydrophobicity scale defined by Kyttle and Doolittle (“A simple method for displaying the hydropathic character of a protein” J Mol Biol. 1982 May 5;157(1):105-32.PMID: 7108955 DOI: 10.1016/0022-2836(82)90515-0). On the right, averaged-hydrophobicity index of proteins ─ divided into 100 bins ─ that were either encoded by MAPKi-upregulated mRNAs (in red) or by MAPKi-downregulated mRNAs (in blue). The red- and blue-background represent the variability (standard deviation) of the hydrophobicity index insight each of the two protein groups. The bottom black lines represent the statistical analysis (two-tailed t-test FDR ≤ 0.05) computed at each position.
b Distribution curves of the proportion (density) of proteins encoded by MAPKi-upregulated mRNAs (red) or by MAPKi-downregulated mRNAs (blue) as a function of the frequency of Lys, Glu, Asp, Trp, Phe or Leu. Shifting the curves to the right means that a higher proportion of mRNAs in a given mRNA population has a higher frequency of a given amino acid.
c Relative frequency of codons in MAPKi-downregulated mRNAs and MAPKi-upregulated mRNAs when compared to the frequency of codons in A375-expressed mRNAs. ***FDR < 0.001, **FDR ≤ 0.01, *FDR ≤  0.05 (beta regression analysis followed by a Tukey’s test [pairwise comparison]).

## Slide 2
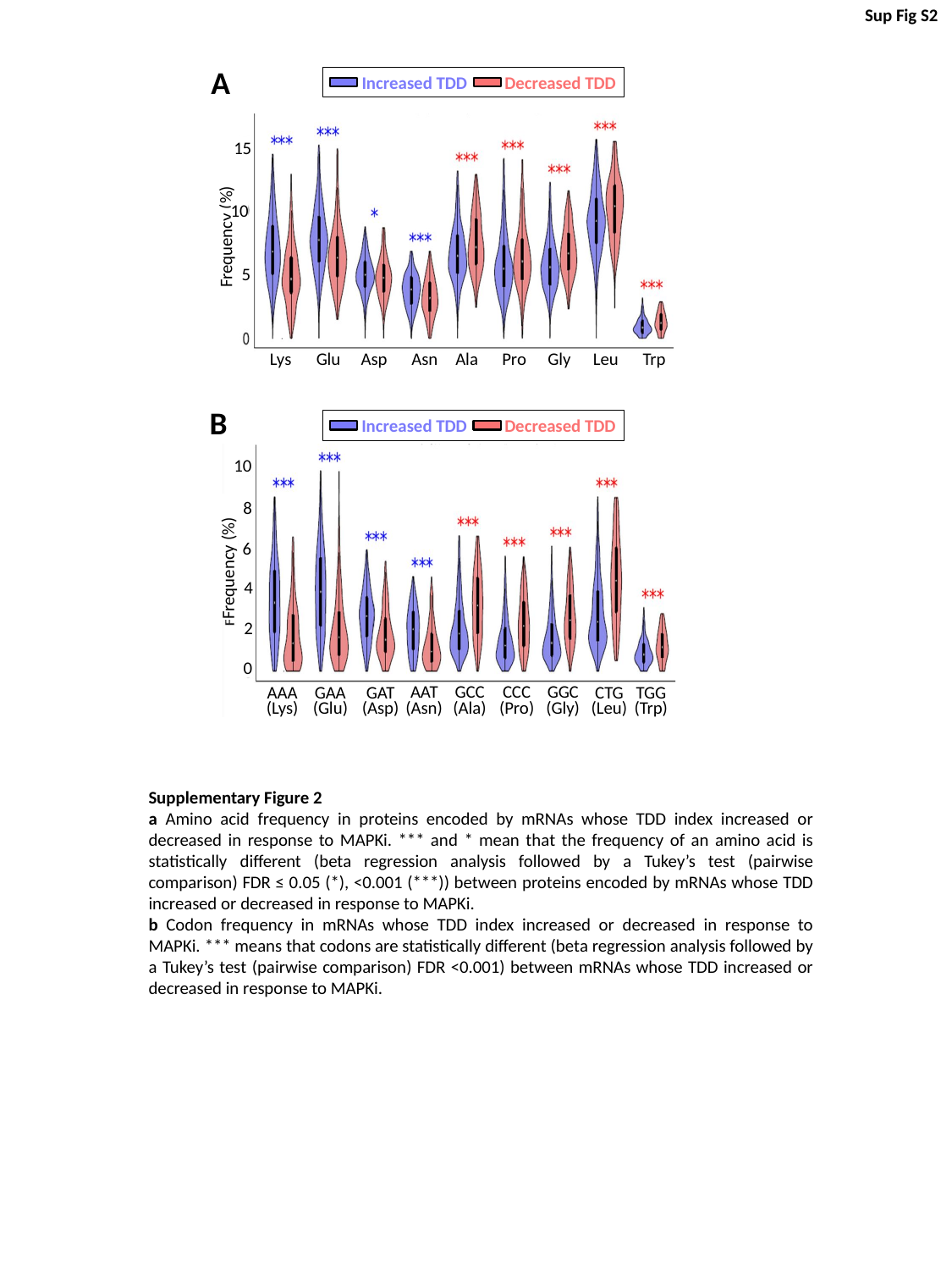

Sup Fig S2
A
Increased TDD
Decreased TDD
15
10
Frequency (%)
 5
Ala
Pro
Gly
Leu
Trp
Lys
Glu
Asp
Asn
B
10
8
6
Frequency (%)
4
2
0
Increased TDD
Decreased TDD
AAT
(Asn)
GCC
(Ala)
CCC
(Pro)
GGC
(Gly)
AAA
(Lys)
GAA
(Glu)
GAT
(Asp)
CTG
(Leu)
TGG
(Trp)
Asn
Supplementary Figure 2
a Amino acid frequency in proteins encoded by mRNAs whose TDD index increased or decreased in response to MAPKi. *** and * mean that the frequency of an amino acid is statistically different (beta regression analysis followed by a Tukey’s test (pairwise comparison) FDR ≤ 0.05 (*), <0.001 (***)) between proteins encoded by mRNAs whose TDD increased or decreased in response to MAPKi.
b Codon frequency in mRNAs whose TDD index increased or decreased in response to MAPKi. *** means that codons are statistically different (beta regression analysis followed by a Tukey’s test (pairwise comparison) FDR <0.001) between mRNAs whose TDD increased or decreased in response to MAPKi.

## Slide 3
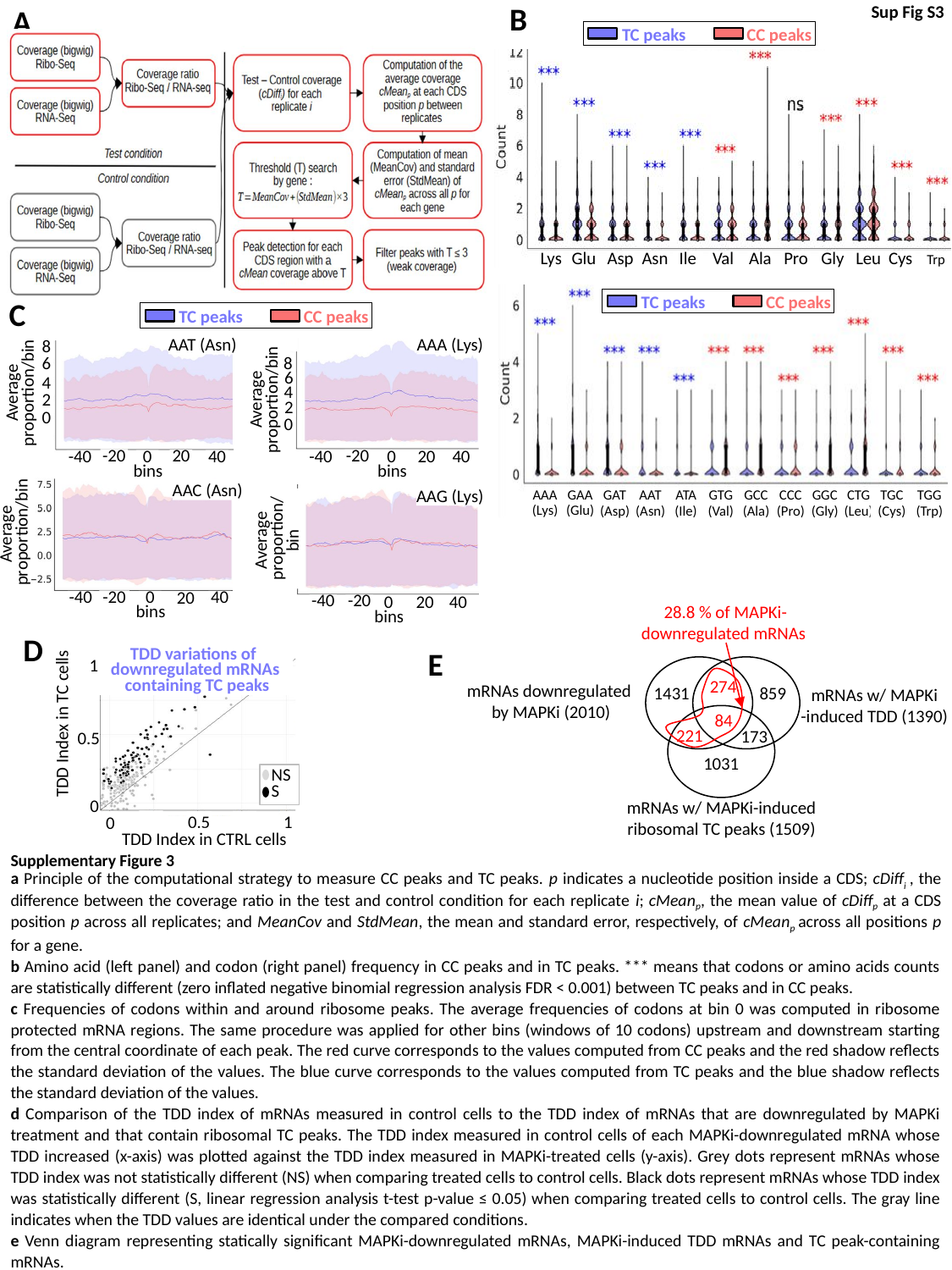

Sup Fig S3
B
Ile
Val
Ala
Pro
Gly
Leu
Cys
Trp
Lys
Glu
Asp
Asn
A
TC peaks
CC peaks
AAA
(Lys)
GAA
(Glu)
GAT
(Asp)
AAT
(Asn)
ATA
(Ile)
GTG
(Val)
GCC
(Ala)
CCC
(Pro)
GGC
(Gly)
CTG
(Leu)
TGC
(Cys)
TGG
(Trp)
TC peaks
CC peaks
C
AAT (Asn)
AAA (Lys)
8
6
8
6
Average
proportion/bin
4
Average
proportion/bin
4
2
2
0
0
-20
20
0
40
-40
-20
-40
20
0
40
bins
bins
AAC (Asn)
AAG (Lys)
Average
proportion/bin
Average
proportion/bin
-20
-40
0
40
20
-40
-20
20
0
40
bins
bins
TC peaks
CC peaks
28.8 % of MAPKi- downregulated mRNAs
D
TDD variations of downregulated mRNAs
 containing TC peaks
TDD Index in TC cells
0.5
NS
S
0
1
0.5
0
E
1
mRNAs downregulated
 by MAPKi (2010)
mRNAs w/ MAPKi
-induced TDD (1390)
274
1431
859
84
221
173
1031
mRNAs w/ MAPKi-induced
ribosomal TC peaks (1509)
TDD Index in CTRL cells
Supplementary Figure 3
a Principle of the computational strategy to measure CC peaks and TC peaks. p indicates a nucleotide position inside a CDS; cDiffi , the difference between the coverage ratio in the test and control condition for each replicate i; cMeanp, the mean value of cDiffp at a CDS position p across all replicates; and MeanCov and StdMean, the mean and standard error, respectively, of cMeanp across all positions p for a gene.
b Amino acid (left panel) and codon (right panel) frequency in CC peaks and in TC peaks. *** means that codons or amino acids counts are statistically different (zero inflated negative binomial regression analysis FDR < 0.001) between TC peaks and in CC peaks.
c Frequencies of codons within and around ribosome peaks. The average frequencies of codons at bin 0 was computed in ribosome protected mRNA regions. The same procedure was applied for other bins (windows of 10 codons) upstream and downstream starting from the central coordinate of each peak. The red curve corresponds to the values computed from CC peaks and the red shadow reflects the standard deviation of the values. The blue curve corresponds to the values computed from TC peaks and the blue shadow reflects the standard deviation of the values.
d Comparison of the TDD index of mRNAs measured in control cells to the TDD index of mRNAs that are downregulated by MAPKi treatment and that contain ribosomal TC peaks. The TDD index measured in control cells of each MAPKi-downregulated mRNA whose TDD increased (x-axis) was plotted against the TDD index measured in MAPKi-treated cells (y-axis). Grey dots represent mRNAs whose TDD index was not statistically different (NS) when comparing treated cells to control cells. Black dots represent mRNAs whose TDD index was statistically different (S, linear regression analysis t-test p-value ≤ 0.05) when comparing treated cells to control cells. The gray line indicates when the TDD values are identical under the compared conditions.
e Venn diagram representing statically significant MAPKi-downregulated mRNAs, MAPKi-induced TDD mRNAs and TC peak-containing mRNAs.

## Slide 4
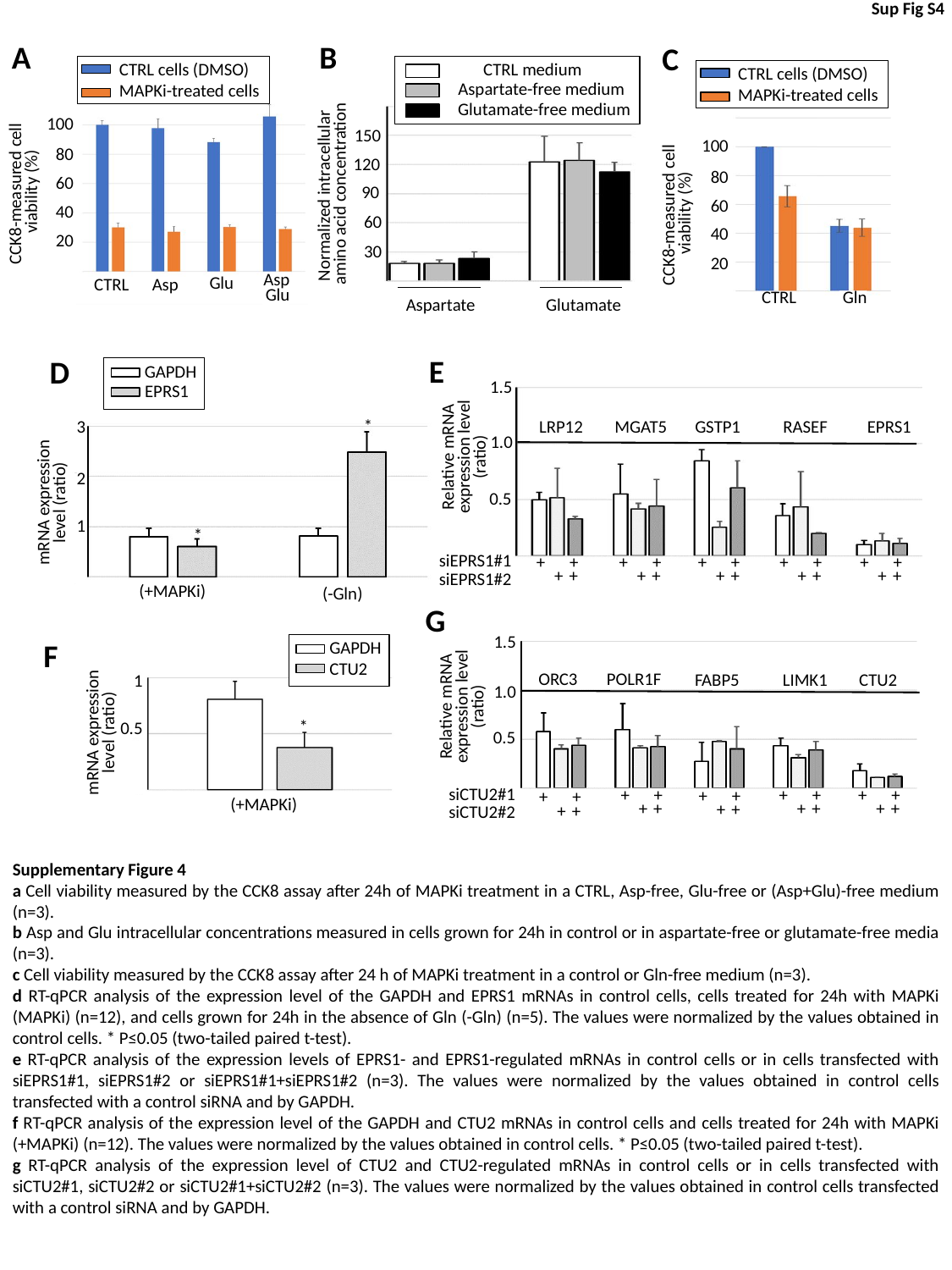

Sup Fig S4
A
B
C
CTRL cells (DMSO)
MAPKi-treated cells
CTRL medium
Aspartate-free medium
Glutamate-free medium
CTRL cells (DMSO)
MAPKi-treated cells
100
150
100
80
120
80
60
CCK8-measured cell
 viability (%)
Normalized intracellular
amino acid concentration
90
CCK8-measured cell
 viability (%)
60
40
60
40
20
30
20
Asp
Glu
Glu
CTRL
Asp
Glutamate
Aspartate
Gln
CTRL
E
D
GAPDH
EPRS1
1.5
1.0
0.5
*
LRP12
MGAT5
GSTP1
RASEF
EPRS1
3
Relative mRNA expression level (ratio)
2
mRNA expression level (ratio)
*
1
siEPRS1#1
+
+
+
+
+
+
+
+
+
+
+
+
+
+
+
+
+
+
+
+
siEPRS1#2
(+MAPKi)
(-Gln)
G
GAPDH
CTU2
1.5
1.0
0.5
F
ORC3
POLR1F
FABP5
LIMK1
CTU2
1
Relative mRNA expression level (ratio)
*
mRNA expression level (ratio)
0.5
siCTU2#1
+
+
+
+
+
+
+
+
+
+
+
+
+
+
+
+
+
+
+
+
(+MAPKi)
siCTU2#2
Supplementary Figure 4
a Cell viability measured by the CCK8 assay after 24h of MAPKi treatment in a CTRL, Asp-free, Glu-free or (Asp+Glu)-free medium (n=3).
b Asp and Glu intracellular concentrations measured in cells grown for 24h in control or in aspartate-free or glutamate-free media (n=3).
c Cell viability measured by the CCK8 assay after 24 h of MAPKi treatment in a control or Gln-free medium (n=3).
d RT-qPCR analysis of the expression level of the GAPDH and EPRS1 mRNAs in control cells, cells treated for 24h with MAPKi (MAPKi) (n=12), and cells grown for 24h in the absence of Gln (-Gln) (n=5). The values were normalized by the values obtained in control cells. * P≤0.05 (two-tailed paired t-test).
e RT-qPCR analysis of the expression levels of EPRS1- and EPRS1-regulated mRNAs in control cells or in cells transfected with siEPRS1#1, siEPRS1#2 or siEPRS1#1+siEPRS1#2 (n=3). The values were normalized by the values obtained in control cells transfected with a control siRNA and by GAPDH.
f RT-qPCR analysis of the expression level of the GAPDH and CTU2 mRNAs in control cells and cells treated for 24h with MAPKi (+MAPKi) (n=12). The values were normalized by the values obtained in control cells. * P≤0.05 (two-tailed paired t-test).
g RT-qPCR analysis of the expression level of CTU2 and CTU2-regulated mRNAs in control cells or in cells transfected with siCTU2#1, siCTU2#2 or siCTU2#1+siCTU2#2 (n=3). The values were normalized by the values obtained in control cells transfected with a control siRNA and by GAPDH.
